# An innovation for improving maternal, newborn and child health (MNCH) service delivery in Jigawa State, northern Nigeria: a qualitative study of stakeholders’ perceptions about clinical mentoring

**DOI:** 10.1186/s12913-015-0724-4

**Published:** 2015-02-15

**Authors:** Ekechi Okereke, Jamilu Tukur, Amina Aminu, Jean Butera, Bello Mohammed, Mustapha Tanko, Ibrahim Yisa, Benson Obonyo, Mike Egboh

**Affiliations:** Abt Associates Nigeria, Partnership for Transforming Health Systems Phase 2, (PATHS2), Abuja, Nigeria; Department of Obstetrics and Gynaecology, Bayero University/Aminu Kano Teaching Hospital, Kano, Nigeria

**Keywords:** Clinical mentoring, Innovation, Service delivery, Qualitative research, Northern Nigeria

## Abstract

**Background:**

An effective capacity building process for healthcare workers is required for the delivery of quality health care services. Work-based training can be applied for the capacity building of health care workers while causing minimum disruption to service delivery within health facilities. In 2012, clinical mentoring was introduced into the Jigawa State Health System through collaboration between the Jigawa State Ministry of Health and the Partnership for Transforming Health Systems Phase 2 (PATHS2). This study evaluates the perceptions of different stakeholders about clinical mentoring as a strategy for improving maternal, newborn and child health service delivery in Jigawa State, northern Nigeria.

**Methods:**

Interviews were conducted in February 2013 with different stakeholders within Jigawa State in Northern Nigeria. There were semi-structured interviews with 33 mentored health care workers as well as the health facility departmental heads for Obstetrics and Pediatrics in the selected clinical mentoring health facilities. In-depth interviews were also conducted with the clinical mentors and two senior government health officials working within the Jigawa State Ministry of Health. The qualitative data were audio-recorded; transcribed and thematically analysed.

**Results:**

The study findings suggest that clinical mentoring improved service delivery within the clinical mentoring health facilities. Significant improvements in the professional capacity of mentored health workers were observed by clinical mentors, heads of departments and the mentored health workers. Best practices were introduced with the support of the clinical mentors such as appropriate baseline investigations for pediatric patients, the use of magnesium sulphate and misoprostol for the management of eclampsia and post-partum hemorrhage respectively. Government health officials indicate that clinical mentoring has led to more emphasis on the need for the provision of better quality health services.

**Conclusion:**

Stakeholders report that the introduction of clinical mentoring into the Jigawa State health system gave rise to an improved capacity of the mentored health care workers to deliver better quality maternal, newborn and child health services. It is anticipated that with a scale up of clinical mentoring, health outcomes will also significantly improve across northern Nigeria.

## Background

Increasing the number of skilled health workers to provide quality health services is a key health systems strengthening objective [1]. Within the health system, capacity building aims to strengthen skills, knowledge, resources and existing infrastructures in order to more effectively tackle challenges or problems [2]. Capacity building of healthcare workers beyond their formal training is crucial for effective health care service delivery [3]. There are training programs which involve the training of one or more individuals from a health care facility [4], however the effective application of the knowledge and skills learnt during trainings involves multidisciplinary teams working together to deliver healthcare services [5]. Bringing health workers for centralized trainings tend to disrupt service delivery in health facilities and this could have serious consequences for vulnerable and underserved populations [6]. These facts highlight the need for alternative approaches which address capacity building challenges and improve health care service delivery especially within developing countries.

Within clinical practice in particular, work based training is a key part of professional development which has the capacity to develop health care workers in ways quite different from, but complementary to formal *classroom* training [7]. The key objective of work based training is knowledge and skills transfer while causing minimum disruption to work processes [8,9]. Clinical mentoring, a form of work-based training, should start as soon as formal training ends and take place at health facilities where health workers treat as well as manage patients. A clinical mentor is usually a clinical practitioner e.g. a doctor or nurse with relevant experience, knowledge and skills that are transferable for the professional development of other less experienced health care workers [10,11]. Clinical mentoring has been applied in resource-constrained settings to improve access to and scale up of HIV care and antiretroviral therapy [10]. A typical capacity building and clinical mentoring conceptual framework among health workers should include a mixture of linear, top-down, bottom-up and expert-led planning and learning processes [12].

Nigeria has an estimated population of over 140 million people [13]. However evidence indicates that maternal, newborn and child mortality statistics are particularly poor across northern Nigeria [14]. Maternal mortality rates for northern Nigeria have been estimated to be as high as 1,271 maternal deaths per 100,000 live births in four states of northern Nigeria i.e. Jigawa, Katsina, Yobe, and Zamfara States [15]. A recent study reports under-5 mortality rates (U5MR) for some States in northern Nigeria as 160 deaths per 1,000 live births which is higher than the national figure of 157 deaths per 1,000 live births [16]. These statistics highlight a maternal and child health crisis, especially across northern Nigeria which needs urgent attention and response.

There is considerable evidence that work-based training approaches such as clinical mentoring has been successfully utilized to transfer knowledge and skills in several fields such as management of HIV/AIDS [17], neonatal care [18] and family planning [19]. Clinical mentoring was introduced as a pilot intervention in Jigawa State where the ratio of health workers per 100,000 of population is significantly lower than the national average for most cadres of health workers. The acute shortage of healthcare workers and existing lack of adequate capacity particularly within northern Nigeria necessitates the adoption of interventions to bridge the prevailing human resources for health gaps. Within northern Nigeria, in addition to requiring more healthcare workers there is need for *continuous* professional capacity development to effectively provide the right quality of healthcare services to improve maternal, newborn and child health outcomes. It was hypothesized that the implementation of clinical mentoring would assist in addressing the prevailing challenges to maternal, newborn and child healthcare service delivery within northern Nigeria. The objective of this study was to elicit the perceptions of stakeholders about clinical mentoring and its potential for improving maternal, newborn and child health service delivery within Jigawa State in northern Nigeria. The study highlights some successes as well as some challenges associated with the implementation of clinical mentoring within the context of the Jigawa State health care system.

## Methods

### Study setting

Jigawa State is within the north western part of Nigeria and consists of 27 Local Government Areas [20]. According to the 2006 census, the State has a total population of about 4,348,649 inhabitants [21] with Hausa and Fulani as the predominant ethnic groups. The State’s health system replicates the WHO-recommended “District Health System” which is a system used by many developing countries to ensure the provision of quality health services to individuals. This approach integrates the health system, bringing both primary and secondary care services under one management structure [22]. In Jigawa State, this district health system is called the ‘Gunduma health system’ and it is managed by the Gunduma Health Board, an affiliate of the Jigawa State Ministry of Health. However the health service statistics in Jigawa State mirrors the situation in most of northern Nigeria. Maternal mortality rates as high as 2284 deaths per 100,000 live births have been reported from a tertiary health center located within rural settings in Jigawa State [23]. Table 1 outlines the list of health facilities and cadre of health workers which participated in the clinical mentoring intervention with the estimated catchment population and location i.e. Local Government Area (LGA) within Jigawa State.

**Table 1:** **List of health facilities and cadre of health workers per health facility participating in clinical mentoring in Jigawa State**

**S/N**	**Name of health facility**	**Cadre of health workers**	**Classification of health facility**	**Estimated catchment population**	**Local Government Area (LGA)**
1	Hadejia General Hospital	Nurse/Midwife	CEOC	104, 286	Hadejia
		Nurse/Midwife			
		Nurse/Midwife			
		Nurse/Midwife			
		Nurse/Midwife			
		CHEW			
		CHEW			
		CHEW			
2	Ringim General Hospital	Doctor	CEOC	18, 609	Ringim
		Nurse/Midwife			
		Nurse/Midwife			
		Nurse/Midwife			
		Nurse/Midwife			
		Nurse/Midwife			
		Nurse/Midwife			
		CHEW			
3	Garki PHC	Doctor	BEOC	21, 118	Garki
		Nurse/Midwife			
		Nurse/Midwife			
		CHEW			
		CHEW			
		CHEW			
4	Gwaram Cottage Hospital	Doctor	BEOC	10, 412	Gwaram
		Nurse/Midwife			
		Nurse/Midwife			
		Nurse/Midwife			
		CHEW			
		CHEW			
5	Basirka PHC	Doctor	PHC	7, 250	Gwaram
		Nurse/Midwife			
		Nurse/Midwife			
		CHEW			
		CHEW			

Key: CEOC: Comprehensive Emergency Care Centre; BEOC: Basic Emergency Obstetric care Centre; PHC: Primary Health Centre.

CHEW: Community Health Extension Worker.

### The clinical mentoring intervention within Jigawa State, northern Nigeria

In July 2012, clinical mentoring was introduced in selected health facilities in Jigawa State within northern Nigeria. The clinical mentoring intervention involved once weekly visits to five selected health facilities by three consultant obstetricians and three consultant pediatricians. The pilot clinical mentoring intervention was designed such that each of the five intervention health facilities was visited by a consultant obstetrician as well as a consultant pediatrician. Thirty three (33) health workers to be mentored *were randomly selected and recruited* to participate in clinical mentoring from different cadres of health workers that routinely work in the five selected health facilities in Jigawa State (please see Table 1). The mentees were junior medical officers, nurses, midwives, community health officers and community health extension workers (CHEWs) working in the participating health facilities. The rationale for selecting health workers from different cadres to participate in the clinical mentoring intervention was to include cadres of health workers providing *clinical healthcare services* to patients attending the health facilities. During their visits, the clinical mentors performed activities such as teaching during ward rounds, carrying out surgeries, as well as conducting outpatient clinics. The clinical mentors also organize meetings such as maternal death review meetings as well as clinical seminars on relevant topics on maternal, newborn and pediatric care. There were *no specific pairing* of clinical mentors to particular health workers for mentoring but clinical mentors who visited each health facility mentored all the health workers recruited to take part in clinical mentoring within each health facility.

The clinical mentors were identified through the professional network of PATHS2’s consultant on clinical mentoring. The clinical mentors work full time at teaching hospitals in the surrounding States of Kano and Bauchi States. These clinical mentors *were recruited through purposive sampling* from tertiary health facilities i.e. the teaching hospitals and were allowed to travel from their places of primary assignment to the clinical mentoring health facilities. The clinical mentoring intervention involves collaboration between the Jigawa State Ministry of Health (which owns and manages the intervention health facilities) and the Partnership for Transforming Health Systems Phase 2 (PATHS2) which provides support for the allowances of the clinical mentors. PATHS2 is a health systems strengthening project sponsored by United Kingdom’s Department for International Development (DFID) and implemented by a consortium of partners led by Abt Associates. PATHS2 works in collaboration with the Government of Nigeria (at Federal and State level) and other stakeholders to improve the planning, financing and delivery of sustainable health services to Nigerians.

### Data collection and analysis

Each of the different subgroups of study participants (clinical mentors, mentored health workers, health facility departmental in-charges of Obstetrics/Pediatrics and Jigawa State Government health officials) were identified as stakeholders in the implementation of clinical mentoring and regarded as key informants for the study. In February 2013, interviews by the study team (led by the first two authors of this paper) were conducted with the stakeholders of the clinical mentoring intervention in Jigawa State. The interviews were documented using tape recorders. The qualitative data were transcribed, a pre-determined coding framework was applied and subsequently thematic analyses of the data were undertaken. Members of the study team who were involved during the data collection process also provided assistance in identifying relevant themes. Regular meetings were held by the study team in order to reach consensus on the interpretation of the data collected during the interviews.

A qualitative approach was adopted for this study so as to provide detailed information and sufficiently elicit the opinions, experiences as well as perceptions of the different stakeholders with respect to the clinical mentoring intervention as opposed to a quantitative approach. The expectation of the study team was that any connections or contradictions between activities and perceived changes arising from the implementation of clinical mentoring will be adequately highlighted using a largely qualitative methodology.

The interview questions were largely tailored to the different stakeholders. The study team carried out in-depth interviews with the clinical mentors to obtain detailed information about their experiences during the implementation of clinical mentoring in participating health facilities. The interview questions for the clinical mentors sought to elicit information about specific changes/improvements achieved as clinical mentors; challenges which were encountered as well as suggestions for improving the current clinical mentoring approach. Key informant interviews with the health facility departmental in-charges (Obstetrics & Pediatrics) were carried out using semi-structured interview guides to elicit their opinions and experiences vis-à-vis the implementation of clinical mentoring within the intervention health facilities. The interview questions for the departmental in-charges sought to obtain information about the activities carried out by the clinical mentors during their visits, benefits of clinical mentoring to the health facility and any improvements observed due to the implementation of clinical mentoring within the intervention health facilities. There were also in-depth interviews with Jigawa State Government health officials to get their perspectives on the effectiveness *or otherwise* of the clinical mentoring intervention within the context of the State-wide health system. The Jigawa State Government health officials were asked questions about the most challenging areas within the Jigawa State health system and how clinical mentoring has helped to address these challenges. Other questions were intended to assess the key lessons learnt, best practices, specific improvements that have been recorded as well as recommendations to improve the implementation of clinical mentoring within the State. The study team also organized structured interviews with the mentored health workers. The structured interviews included questions on the perceived effect/impact of clinical mentoring on health facility activities (please see Table 2).

**Table 2:** **Mentored health workers’ perceptions about the impact of clinical mentoring on health facility activities**

**S/N**	**Health facility activity**	**Perception of level of impact**	**N (%)**
1.	Teaching during ward rounds	Very significant	16 (48.49%)
		Significant	11 (33.33%)
		Average	4 (12.12%)
		Negligible	1 (3.03%)
		Zero	1 (3.03%)
2.	One on one teaching of health workers by the mentor in the health facility	Very significant	10 (30.30%)
		Significant	13 (39.40%)
		Average	8 (24.24%)
		Negligible	2 (6.06%)
		Zero	0 (0.00%)
3.	Clinical seminars on relevant maternal and child health care topics/services	Very significant	8 (24.24%)
		Significant	8 (24.24%)
		Average	6 (18.18%)
		Negligible	3 (9.10%)
		Zero	8 (24.24%)
4.	Mortality/death review	Very significant	12 (36.36%)
		Significant	8 (24.24%)
		Average	6 (18.18%)
		Negligible	3 (9.10%)
		Zero	4 (12.12%)
5.	Outpatient clinics	Very significant	14 (42.43%)
		Significant	10 (30.30%)
		Average	7 (21.21%)
		Negligible	1 (3.03%)
		Zero	1 (3.03%)
6.	Assessing individual patient care/management and planning further action e.g. referrals	Very significant	17 (51.52%)
		Significant	11 (33.33%)
		Average	3 (9.10%)
		Negligible	1 (3.03%)
		Zero	1 (3.03%)
7.	Integrated supportive supervision	Very significant	11 (33.33%)
		Significant	17 (51.52%)
		Average	4 (12.12%)
		Negligible	0 (0.00%)
		Zero	1 (3.03%)
8.	Use of standard clinical guidelines/protocols	Very significant	11 (33.33%)
		Significant	14 (42.43%)
		Average	4 (12.12%)
		Negligible	2 (6.06%)
		Zero	2 (6.06%)

### Ethical approval

Ethical clearance for the study was obtained from the Jigawa State Ministry of Health. In addition, informed consent was obtained from all the study respondents before the interviews commenced. The verbal consent of the study respondents were documented using tape recorders. The objective of the study, respondents’ right to refuse participation and freedom to terminate participation in the study at any time was discussed with each study participant. Study respondents were assured of the confidentiality of their responses.

## Results

### Perspectives of Jigawa State government officials working in the Gunduma health system

Two senior level healthcare officials of the Jigawa State Ministry of Health were interviewed as part of the evaluation of the clinical mentoring intervention within selected health facilities in Jigawa State. Government officials acknowledge that the human resources for health system within the State have multifarious challenges, including but not limited to the following:

i. Inadequate numbers and capacity of the health workforce to provide adequate health care services to the State’s population.
ii. Lack of skilled health care professionals and inadequate training of the health workers within the State.
iii. Attrition of key healthcare workers, including doctors and midwives.

With the introduction of clinical mentoring visits, some of these challenges are being addressed as there appears to be an increase in the number of better trained health professionals providing health services to patients. These improved professional healthcare services have been provided through the expertise and contributions of the clinical mentors. The Gunduma Health System officials indicated that one of the key lessons learnt from the implementation of the clinical mentoring programme was the increased focus the mentoring visits have given to maternal, newborn and child health issues within the health care delivery system of the State. It was also reported that the clinical mentoring intervention has increased awareness about the need to explore other innovative approaches/strategies to address the critical health workers’ situation within Jigawa State. A Gunduma health official remarked:

*“…perhaps a key outcome of the implementation of clinical mentoring will be that Jigawa State better understands other ways to tackle the human resources for health situation and improve healthcare service delivery.”*

Highlighting some challenges however, the Gunduma healthcare officials pointed out that the current approach whereby the clinical mentors visit health facilities, provide services and travel back on the same day naturally limits the time spent providing services and this invariably affects the quality of healthcare services provided. The financial implication of retaining and recruiting more clinical mentors is also a serious challenge and Jigawa State needs to make adequate budgetary allocations to address this challenge.

### Suggestions by the Gunduma health systems officials to improve the clinical mentoring intervention

The key suggestion from the Gunduma health systems officials is that clinical mentoring needs to be effectively integrated into the existing district health system within the State. This integration it was argued would ensure sustainability, greater ownership by the State government and could potentially give rise to more benefits to the State’s health system. In connection with this suggestion, a Gunduma health official remarked:

*“The main suggestion I have is on integrating clinical mentoring into the district health system within the State so that it works through an existing system.”*

It was proposed that adequate temporary accommodation should be provided to enable clinical mentors to follow up on some cases as maybe appropriate. It was also argued that with the perceived increase in patient flow within the mentoring health centres, clinical mentors should spend more visiting hours and days providing services within their assigned health facilities or more clinical mentors should be recruited. Jigawa State needs to have a long term plan of developing a pool of consultants to sustain the clinical mentoring programme.

### Perspectives of the mentored health workers

Thirty three (33) mentored health workers recruited across the five selected health facilities were interviewed as part of the study. Table 2 highlights the perceptions of mentored health workers about the level of impact of clinical mentoring on a set of health facilities activities within the intervention health facilities. The results show that almost half (~48.5%) of the mentored health workers indicated that the clinical mentoring intervention has a ‘very significant’ impact on teaching of health workers during ward rounds. Over 40% as well as about 30% of the mentored health workers respectively reported that clinical mentoring has ‘very significant’ and ‘significant’ impact on the quality of outpatient clinics within the intervention health facilities. Over half of the respondents (~51.5%) indicated that the clinical mentoring intervention has a ‘very significant’ impact on health workers’ ability to assess individual patient’s care/management and subsequently plan for further action. Over half (51.5%) of the mentored health workers indicated that clinical mentoring has a ‘significant’ impact on integrated supportive supervision (ISS) conducted within the intervention health facilities. About one-third of the mentored health workers mentioned that clinical mentoring has a ‘very significant’ impact on the use of standard clinical guidelines and protocols within the intervention health facilities. To summarize the perceptions of the mentored health workers within the intervention health facilities about the effectiveness of the clinical mentoring intervention on their ability to deliver better quality health services, a mentored health worker simply remarked:

*“…my capacity has been built and I have gained in experience.”*

### Perspectives of the clinical mentors

#### Obstetrics clinical mentors

The three obstetrics clinical mentors recruited into the clinical mentoring intervention were interviewed as part of the study. The obstetrics clinical mentors indicated that the preliminary assessment which was carried out at the start of the clinical mentoring intervention provided guidance and thus assisted in identifying weaknesses as well as specific areas for improvement among health workers working within the obstetrics department. Significant changes arising from the clinical mentoring intervention include updated treatment guidelines on basic emergency obstetric health care, pre-eclampsia and eclampsia, post-partum hemorrhage as well as the management of the third stage of labour. An obstetrics clinical mentor remarked:

*“We as mentors have introduced clinical protocols to guide the health workers in the conduct of their work e.g. protocols for magnesium sulphate, management of post-partum hemorrhage, and the management of the third stage of labour…”*

Clinical mentors report that as a result of the mentoring visits, infection prevention practices have been introduced into the health facilities where they visit. Such emphasis on infection prevention will tend to minimize the possibility of patients picking up ‘hospital-based infections’ while visiting the clinical mentoring health facilities.

#### Pediatrics clinical mentors

The three pediatrics clinical mentors participated in interviews as part of the evaluation of the clinical mentoring intervention. The pediatrics clinical mentors indicated that standard treatment guidelines for the management of different pediatric cases have been introduced following the mentoring visits. A pediatric clinical mentor enthused:

*“In health facilities in which we visit, we have developed and introduced treatment protocols, some of which are on the notice boards in some wards…”*

There are other significant changes attributable to clinical mentoring as highlighted by a pediatric clinical mentor:

*“We have introduced the use of nasogastric (NG) tubes for feeding pediatric patients with severe malnutrition”.*

Another pediatric clinical mentor pointed out:

*“The practice of weighing pediatric patients during each visit which was not the case before the start of the clinical mentoring intervention has also been introduced”.*

This approach of measuring pediatric patients during each visit has most likely given rise to the appropriate calculation of dosages for pediatric patients in addition to effective growth monitoring as part of best practices towards managing pediatric cases. A pediatric clinical mentor indicated:

*“We have made significant efforts within the mentoring centers to ensure that weighing scales were acquired for use within the pediatrics department”.*

Greater emphasis has also been placed on better record keeping by the health care workers. Alluding to the changes arising from the mentoring visits, a pediatrics clinical mentor remarked:

*“A good part of the mentoring visits for me is seeing the improvement in the mentee’s knowledge, skills and confidence…”*

### Suggestions by the clinical mentors for the improvement of the clinical mentoring intervention

As a way to improve the effectiveness of clinical mentoring, a clinical mentor suggested that it would be more appropriate *to assign one mentor to cover a health facility* and not one mentor covering more than one health facility as is currently the case. This will ensure that the clinical mentor concentrates on the cases and health workers to be mentored in just one assigned health facility. Another suggestion was that temporary accommodation should be provided for the clinical mentors to allow the mentors follow up on cases when and where necessary. The current set-up whereby mentors travel from their primary location, provide services and have to travel back the same day limits the quality *and quantity* of care provided to patients. It is also not advantageous to the patients who may need further monitoring such as those that have had surgeries.

It was proposed that payment incentives could be introduced to increase the motivation of the clinical mentors. Furthermore, it was recommended that the benefits of the mentoring visits should be extended beyond the health workers in the mentoring centres, such that health workers working in surrounding health facilities could be invited on the days that the consultants visit to learn alongside the health workers in the mentoring centres. In addition, there should be proper community mobilization such that the communities within the catchment areas of the health facilities should be enlightened about the visit of the consultants to ensure that a larger proportion of community members access and utilize the services of the visiting consultants. A clinical mentor remarked:

*“There is need to create more opportunities and sensitize community leaders in the surrounding communities while also liaising with the health facility in-charges to assist in mobilizing community members so as to better utilize the expertise and services offered by the clinical mentors.”*

### Perspectives of the health workers in-charge of obstetrics in the intervention health facilities

Healthcare workers in-charge of obstetrics in each of the five intervention health facilities took part in interviews as part of the evaluation of the implementation of clinical mentoring. Healthcare workers in-charge of obstetrics indicated that clinical mentors have introduced new skills and updated the knowledge of the mentored health workers working in obstetrics:

*“The clinical mentors are responsible for the introduction of the use of magnesium sulphate as the standard treatment for pre-eclampsia and eclampsia within the intervention health facilities.”*

In addition, a health worker in-charge of obstetrics in one of the intervention health facilities indicated:

*“There is an improvement in the management of post-partum hemorrhage as mentored health workers were trained on the proper use of misoprostol”.*

Another health worker in-charge of obstetrics pointed out:

*“Proper record keeping and improved charting of medications have also been observed…”*

The health facility departmental in-charges mentioned that health workers appear more confident when handling cases and this was reflected in comments from a health facility departmental in-charge:

*“Caesarean section (C/S) deliveries are more likely to be routinely carried out for some pregnancies especially when there are abnormal conditions that complicate normal vaginal delivery”.*

The obstetrics departments of the intervention health facilities suggest an increase in patient flow as well as a decrease in the number of referrals out of the health facility to higher level health facilities. An in-charge of obstetrics in one of the intervention health facilities pointed out:

*“The clinical mentor is assisting the health facility in addressing high burdens of anaemia and malaria in pregnancy within the communities that utilize the services of the health facility”*

An obstetrics unit head within an intervention health facility also indicated:

*“We now record the expected due date (EDD) for prospective mothers as part of best practices for effective maternal healthcare…and to help us with this the clinical mentor came with a calendar so that the EDD for clients can be easily calculated.”*

### Perspectives of the health workers in-charge of pediatrics in the intervention health facilities

The health workers in-charges of pediatrics department in each of the five clinical mentoring intervention health facilities were interviewed as part of the study. The health workers in-charge of pediatrics concur with the mentored health workers and suggest that there has been an increase in pediatric patients coming to access health services. It was implied by health workers in-charge of pediatrics that the mentored health workers tend to better manage patients, thus likely reducing the number of pediatric cases referred out of the health facilities. A pediatrics unit in-charge remarked:

*“More patients are coming…a health worker at a nearby health facility referred a patient and insisted that the patient visits on the day the clinical mentor visits this health facility…”*

Aligning with the responses from the pediatric clinical mentors, pediatric unit in-charges indicate that some notable best practices have been introduced by the clinical mentors; for e.g. weighing pediatric patients which is crucial for the appropriate calculation of pediatric dosages as well as for growth monitoring. An in-charge of pediatrics in one of the intervention health facilities also pointed out:

*“The clinical mentor introduced the use of ambu-bags and these have been particularly useful for neonatal resuscitation and reducing neonatal mortality”.*

Proper baseline investigations and tests are now more likely to be carried out as part of standard management of pediatric cases following the introduction of clinical mentoring. The pediatrics clinical mentors also introduced changes to the management of malnutrition such as the use of nasogastric (NG) tubes for managing malnourished children within the intervention health facilities. A health worker in-charge of the pediatrics remarked:

*“There have been some changes in the treatment protocols for e.g. malnutrition; we have been taught that we should be careful with the use of IV fluids and instead insert NG-tubes especially for severely malnourished pediatric patients.”*

With the accessibility of the consultant pediatricians to health workers working in the intervention health facilities, it was reported that health workers tend to better manage clinical cases and if there is need, the clinical mentors could be contacted using a mobile telephone and thus the necessary guidance can be provided. This perception of an improved standard of pediatric care within the intervention health facilities was summarized by a health worker in-charge of pediatrics in one of the intervention health facilities as follows:

*“Clinical mentoring has introduced new clinical approaches…resulting in improved standards of health care for our pediatric patients.”*

## Discussion

This study set out to investigate stakeholders’ perceptions of clinical mentoring as an innovation for improving maternal, newborn and child health service delivery within Jigawa State in northern Nigeria. The study explored the implementation of clinical mentoring from four unique perspectives: clinical mentors, mentored health workers, departmental in-charges of Pediatrics and Obstetrics within intervention health facilities as well as government officials from the Gunduma Health Board. Utilizing a qualitative study design, the study elicits the responses of key actors in the implementation of the clinical mentoring intervention, discusses the implications for improving health care service delivery and outlines some key policy recommendations.

Poor performance among health workers is usually associated with the lack of adequate knowledge and skills [1]. However the responses of the clinical mentors, mentored health workers, and Pediatrics/Obstetrics departmental in-charges suggest that the professional capacity of the mentored health workers improved significantly following the implementation of clinical mentoring. This work-based capacity building approach in the long term is expected to promote retention of health workers as well as improve service delivery as suggested elsewhere [24]. Work-based training approaches have been implemented to strengthen professional capacity [25-27] but in this case it was specifically employed to increase health workers’ capacity to improve maternal, newborn and child health service delivery. Sustainability issues need to be addressed as there are legitimate concerns about the long term retention of the knowledge and skills gained by the mentored health workers especially as evidence indicate that knowledge acquired through trainings can possibly deteriorate within a short duration where adequate sustainability mechanisms are lacking [28].

The preliminary assessments conducted at the start of the clinical mentoring intervention provided useful pointers on specific areas to focus on during the capacity building process. The vast majority of mentored health workers suggest that clinical mentoring did not only strengthen health workers capacity but it also appears to have improved service delivery and health facility management within the intervention health facilities. The contributions of clinical mentoring to health facility management and service delivery were also reiterated by the healthcare workers in charge of both Pediatrics and Obstetrics: clinical mentors have utilized their expertise to update the knowledge and skills of mentored health workers. More importantly, best practices were introduced and are being institutionalized with the support of the clinical mentors such as the use of magnesium sulphate for eclampsia management, misoprostol for the management of post-partum hemorrhage, weighing pediatric patients *during each visit* to ensure the appropriate calculation of pediatric dosages, appropriate baseline investigations for pediatric patients as well as the use of *ambu-bags* for neonatal resuscitation to reduce neonatal mortality.

However serious considerations should be paid to the recommendations proposed by the clinical mentors such as the provision of temporary accommodation to allow the mentors follow up on cases when and where necessary. As a solution, the government can provide some form of “doctors’ lodge” to provide overnight accommodation for the clinical mentors to address the challenge of temporary accommodation. Pay for performance incentives can also be introduced to further motivate the clinical mentors which will have direct consequences on the quality of services provided at the health facilities. Criteria for computing these financial incentives could be based for example on the number of complicated cases managed per week or month over and above a set target. Concerted efforts should also be invested to ensure that the benefits of the mentoring visits are fully exploited by the catchment populations of the intervention health facilities through employing effective community mobilization strategies. Community mobilization could involve the use of meetings in surrounding communities that utilize the services of the health facilities as well as the mass media such as radio to inform communities about the availability of expert healthcare in the clinical mentoring health facilities. The availability of more patients through community mobilization could lead to better mentoring because of the exposure of the mentees to a wider variety of clinical cases. In addition, increasing access to the expertise of the clinical mentors by providing mentoring opportunities for the majority of health workers working within as well as others working outside the current mentoring centers should be a priority.

Another obvious recommendation is the scale up of clinical mentoring to health facilities outside the current mentoring centers. In carrying out such a scale up to other health facilities, careful consideration should be paid to a more holistic system strengthening approach to the health system including the provision of essential drugs, adequate financial resource mobilization and reliable health information systems in addition to proper governance and accountability mechanisms. This is important because as implied elsewhere [1], simply scaling up innovative interventions in weak health systems is likely to waste resources and will possibly fail to show the anticipated improvements in health outcomes. Additionally, there has been much effort in the past directed at increasing the number of health workers especially within developing countries but less attention has been paid to developing capacity building models which address specific workplace needs [5]. This clinical mentoring approach ensures that the training of health workers is contextual and is tailored to address the specific needs of the health workers and health facilities. In the short and long term, this capacity building approach is expected to improve service delivery within the health facilities as the clinical mentors will continuously provide professional support that will improve maternal, newborn and child health care services [29].

This study has some key limitations: there is the possibility of response bias particularly among the mentored healthcare workers and clinical mentors. In addition the study design excluded health workers not participating in this clinical mentoring approach but working within the intervention health facilities, who may have provided an entirely different perspective. Furthermore, since the study was conducted just about six months after clinical mentoring commenced, there is lack of evidence on the longer term impact of clinical mentoring on health workers’ capacity and healthcare service delivery.

## Conclusion

This study suggests that the introduction of clinical mentoring into the Jigawa State health system has given rise to mentored health care workers with improved capacity to deliver better quality maternal, newborn and child health services. There is strong optimism that consequently there will be more emphasis placed on the need for and the provision of better maternal, newborn and child healthcare (MNCH) services. However this optimism needs to be matched with organizational support from the health facilities’ management as well as managerial support from the State’s Ministry of Health to improve health workers’ motivation, service delivery and health outcomes. Finally as suggested by healthcare officials working in the Gunduma Health Board, integrating clinical mentoring into the existing district health system within the State will promote ownership by the Government as well as the sustainability of clinical mentoring within the Jigawa State health system.
